# Are Oxidized LDL/β2-glycoprotein I Complexes Pathogenic Antigens in Autoimmune-mediated Atherosclerosis?

**DOI:** 10.1080/10446670410001722186

**Published:** 2004-06

**Authors:** Eiji Matsuura, Luis R. Lopez

**Affiliations:** 1 Department of Cell Chemistry Okayama University Graduate School of Medicine and Dentistry 2-5-1 Shikata-cho Okayama 700-8558 Japan; 2 Corgenix, Inc. Westminster CO 80234 USA

## Abstract

The oxidative modification of low-density lipoprotein (LDL) in the intima of arteries contributes to the initiation and progression of atherosclerotic lesions. We have previously reported that oxidized LDL (oxLDL) interacts with an endogenous plasma protein, β2-glycoprotein I (β2GPI), to form complexes and that the interaction is mediated by oxLDL specific ligands. We have also demonstrated the presence of oxLDL/β2GPI complexes in the blood stream of patients with systemic inflammatory and autoimmune diseases. These findings implicate that oxLDL/β2GPI complexes are possible atherogenic autoantigens. Autoantibodies to oxLDL/β2GPI complexes have been associated with arterial thrombosis. Further, circulating IgG immune complexes containing oxLDL and 2GPI were also detected in patients with systemic lupus erythematosus (SLE) and/or antiphospholipid syndrome (APS). Although many unanswered questions remain about the exact pathogenic mechanism(s) involved, oxLDL/β2GPI complexes may be described as metabolic products relevant in atherogenesis and as significant participants in autoimmune-mediated atherosclerosis.


*Clinical & Developmental Immunology*, June 2004, Vol. 11 (2), pp. 103–111

Taylor & Francis healthsciences

### Are Oxidized LDL/β2-glycoprotein I Complexes Pathogenic Antigens in Autoimmune-mediated Atherosclerosis?

EIJI MATSUURA[a,]* and LUIS R. LOPEZ[b]

[a]*Department of Cell Chemistry, Okayama University Graduate School of Medicine and Dentistry, 2-5-1 Shikata-cho, Okayama 700-8558, Japan;* [b]*Corgenix, Inc., Westminster, CO 80234, USA*

The oxidative modification of low-density lipoprotein (LDL) in the intima of arteries contributes to the initiation and progression of atherosclerotic lesions. We have previously reported that oxidized LDL (oxLDL) interacts with an endogenous plasma protein, β2-glycoprotein I (β2GPI), to form complexes and that the interaction is mediated by oxLDL specific ligands. We have also demonstrated the presence of oxLDL/β2GPI complexes in the blood stream of patients with systemic inflammatory and autoimmune diseases. These findings implicate that oxLDL/β2GPI complexes are possible atherogenic autoantigens. Autoantibodies to oxLDL/β2GPI complexes have been associated with arterial thrombosis. Further, circulating IgG immune complexes containing oxLDL and β2GPI were also detected in patients with systemic lupus erythematosus (SLE) and/or antiphospholipid syndrome (APS). Although many unanswered questions remain about the exact pathogenic mechanism(s) involved, oxLDL/β2GPI complexes may be described as metabolic products relevant in atherogenesis and as significant participants in autoimmune-mediated atherosclerosis.

*Keywords*: Antiphospholipid syndrome; Antiphospholipid antibody; Oxidized LDL; β2-glycoprotein I; Atherosclerosis

### INTRODUCTION

Atherosclerosis is a major health concern of worldwide importance. The experimental evidence has established a causal relationship between atherosclerosis and lipoprotein metabolism. More recently, inflammatory and immunologic mechanisms have been linked to the initiation and progression of atherosclerotic lesions. In particular, the oxidation of low-density lipoprotein (LDL) was identified as an early pro-atherogenic event that promotes the formation of macrophage-derived foam cells (Steinbrecher *et al.*, 1984; Steinberg *et al.*, 1989; Steinberg, 1997; Heinecke, 1997).

The premature occurrence of atherosclerotic lesions associated with significant cardiovascular morbidity and mortality in patients with systemic autoimmune diseases, suggested a contributing role of autoimmunity in the development of atherosclerosis. The antiphospholipid syndrome (APS), frequently diagnosed in the context of an autoimmune disease, is characterized by arterial and venous thromboembolic complications associated with high serum levels of antiphospholipid antibodies (Harris *et al.*, 1983; Hughes *et al.*, 1986). APS is a prothrombotic state likely precipitated by the appearance of anticardiolipin (aCL), lupus anticoagulants (LA) and/or other antiphospholipid antibodies. It is now commonly accepted that the plasma protein β2-glycoprotein I (β2GPI) represents a major antigenic target for antiphospholipid antibodies in patients with APS (McNeil *et al.*, 1990; Galli *et al.*, 1990; Matsuura *et al.*, 1990, 1992, 1994).

Oxidized LDL (oxLDL) is the principal lipoprotein that accumulates within cells of atherosclerotic lesions and it co-localizes with β2GPI (George *et al.*, 1999). These findings further suggested the participation of antiphospholipid antibodies in atherogenesis. In 1993, it was first reported that aCL antibodies from patients with systemic lupus erythematosus (SLE) cross-reacted with malondialdehyde (MDA)-modified LDL (Vaarala *et al.*, 1993). In contrast, it was shown that anti-β2GPI antibodies but not aCL were associated with arterial thrombosis (Tinahones *et al.*, 1998; Romero *et al.*, 1998). We have recently demonstrated that oxLDL binds to endogenous β2GPI and that these complexes (oxLDL/β2GPI) can be found circulating in the blood stream of patients with various chronic diseases, such as SLE, APS, chronic renal disease, diabetes mellitus. Further, IgG immune complexes formed by oxLDL,

*Corresponding author. Tel.: +81-86-235-7402. Fax: +81-86-235-7404. E-mail: eijimatu@md.okayama-u.ac.jp

ISSN 1740-2522 print/ISSN 1740-2530 online © 2004 Taylor & Francis Ltd
DOI: 10.1080/10446670410001722186

β2GPI and anti-β2GPI antibodies have also been detected in SLE and APS patients (Kobayashi *et al.*, 2003). Our recent *in vitro* experiments showed that oxLDL/β2GPI complexes were internalized by macrophages via an anti-β2GPI antibody-mediated phagocytosis (Hasunuma *et al.*, 1997; Kobayashi *et al.*, 2001; Liu *et al.*, 2002; Kobayashi *et al.*, 2003). Thus, circulating oxLDL/β2GPI and/or their IgG immune complexes are most possibly atherogenic. In contrast, recent reports indicated that natural antibodies (mainly of the IgM class) derived from hyperlipidemic mice reduced the incidence of atherosclerosis in experimental models (Chang *et al.*, 1999; Horkko *et al.*, 1999; Binder *et al.*, 2003; Rose and Afanasyeva, 2003).

In this article, we review recent new insights into autoimmune-mediated mechanisms on development of atherosclerosis, mostly based on our recent observations and discuss the possible clinical significance of circulating oxLDL/β2GPI complexes and autoantibodies related to these complexes.

### CURRENT CONCEPTS ON ATHEROGENIC MECHANISMS

Atherosclerosis is a pathological condition in which arteries undergo thickening of the intima causing a decrease in their elasticity. The aorta, coronary and cerebral arteries are blood vessels most commonly affected by atherosclerosis. The appearance of lipid laden foam cells is a characteristic hystologic finding in early atherosclerotic lesions. Figure 1A depicts a current consensus of different events leading to the initial stages of atherosclerosis. Hypercholesterolemia is commonly associated with an elevation of LDL, which is the lipoprotein that accumulates in foam cells. Increasing LDL blood levels together with arterial shear stress may produce a vascular inflammatory response, with the adherence of circulating monocytes to endothelial cells and the migration of these elements (LDL, oxLDL and monocytes) into the intima. The oxidative modification of LDL may be further catalyzed by inflammatory cells at

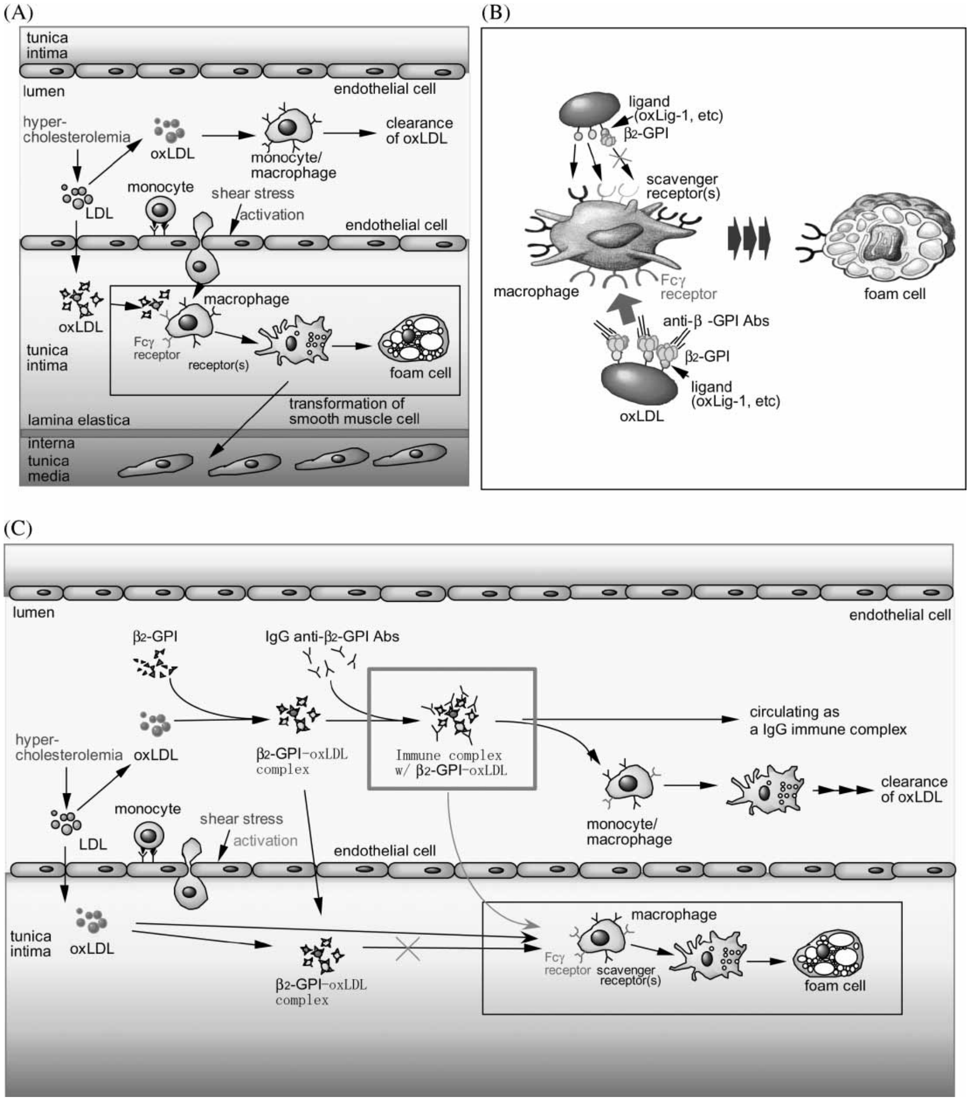

FIGURE 1 Mechanisms of development of atherosclerosis. (A) General consensus of atherogenesis. (B) Possible mechanism of anti-β2GPI autoantibody-mediated oxLDL uptake by macrophages in APS. (C) Proposed mechanism of development of thrombosis in APS.

the site of the arterial lesion, resulting in foam cell formation (oxLDL-loaded macrophages). Numerous pro-inflammatory molecules and/or adhesion molecules also participate in the development of atherosclerosis (Cushing *et al.*, 1990; Rajavashisth *et al.*, 1990; Frostegard *et al.*, 1993; Nakata *et al.*, 1996). In addition, macrophage scavenger receptors and various cell–cell interactions, possibly via CD40 and CD40 ligands, have been reported to be involved in the development of atheroma (Mach *et al.*, 1998). When the endothelial surface of the atherosclerotic lesion becomes damaged and unstable, it may rupture. This event is followed by the activation of blood coagulation mechanisms such as platelet aggregation and thrombi formation, which can result in a complete occlusion of the blood vessel and tissue or organ necrosis, as seen in acute myocardial and cerebral infarction.

### MACROPHAGES AND SCAVENGER RECEPTORS

Macrophages receptors for the specific uptake of LDL were first described by Goldstein *et al.* (1979) and Yamamoto *et al.* (1984). These receptors are down-regulated to prevent lipid overloading. Another type of macrophage receptor was later described for chemically modified-LDLs and named scavenger receptors (Goldstein *et al.*, 1979; Brown and Goldstein, 1985). These scavenger receptors are not down-regulated and may lead to the accumulation of massive amounts of intracellular lipids in macrophages, resulting in the formation of macrophage-derived foam cells. Initially, acetylated-LDL was used as a ligand to study scavenger receptors, but this acetylation was not seen under physiological conditions. In contrast, oxLDL was described as a physiological ligand for scavenger receptors and it was generated by peroxidation of LDL when co-cultured with endothelial cells or when incubated with a metal ion such as $Cu^{2+}$ or $Fe^{2+}$.

Scavenger receptors (i.e. SR-A) were first cloned by Kodama and his colleagues (Kodama *et al.*, 1988; Kodama *et al.*, 1990) and shown to be specific for both acetylated-LDL and $Cu^{2+}$-oxLDL. This was followed by the description of several different types of scavenger receptors, i.e. MARCO (a novel macrophage receptor with collagenous structure), SR-B1, CD36, Macrosialin, CD68, LOX-1, SREC, SRPSOX, etc. (Elomaa *et al.*, 1995; Rigotti *et al.*, 1995; Ramprasad *et al.*, 1995; Sambrano and Steinberg, 1995; Sawamura *et al.*, 1997; Elomaa *et al.*, 1998; Shimaoka *et al.*, 2000; Minami *et al.*, 2001).

### OXIDATIVE-MODIFICATION OF LDL

The LDL particle contains phospholipids, free cholesterols, cholesteryl esters, triglycerides and apolipoprotein B (apoB). Both the lipids and apoB are subjected to oxidation and apoB breaks down into fragments of different sizes (from 14 to over 550 kDa) by oxidative attack, (Fong *et al.*, 1987). A key feature of LDL's oxidation is the breakdown of the polyunsaturated fatty acids to yield a broad array of smaller fragments including aldehydes and ketones that can become conjugated to amino lipids or to apoB (Esterbauer *et al.*, 1987). The polyunsaturated fatty acids in cholesterol esters, phospholipids and triglycerides are subject to free radical-initiated oxidation and can participate in chain reactions that amplify the damage. Recently, two oxidized fatty acid components have been described, 9- or 13-hydroxyoctadecadienoic acid (9-and 13-HODE). These activate peroxisome proliferator-activator receptor γ (PPARγ), a transcriptional regulator of genes linked to lipid metabolism that up-regulate the CD36 scavenger receptor (Nagy *et al.*, 1998). Thus, lipid components of oxLDL generated by PPARγ activation can promote foam cell formation.

Linoleic acid is a predominant polyunsaturated fatty acid in LDL present mainly as a cholesterol ester (Weidtmann *et al.*, 1995). In mild oxLDL, cholesteryl hydroperoxyoctadecadienoic acid (Chol-HPODE) and cholesteryl hydroxyoctadecadienoic acid (Chol-HODE) are the main products of oxidation (Kritharides *et al.*, 1993). It has been reported that Cho-HPODE inactivate platelet-derived growth factor (van Heek *et al.*, 1998). The oxidative breakdown of either free polyunsaturated fatty acids or those esterified at the *sn*-2 position of phospholipids result in fatty acid-hydroperoxides which form highly reactive products containing aldehyde and ketone functions. Such active functions can form Schiff base adducts with lysine residues of the apoB moiety of LDL or other proteins and with primary amine containing phospholipids such as phosphatidylserine and phosphatidylethanolamine.

Cholesterol is also converted to oxysterols and it is especially oxidized at the 7-position. 7-Hydroxycholesterol (both free and esterified) is the major oxysterol formed during early events in LDL oxidation, with 7-ketocholesterol dominating at later stages (Brown *et al.*, 1997). Recent studies indicated that elevated plasma level of 7β-hydroxycholesterol is associated with an increased risk of atherosclerosis (Brown and Jessup, 1999). At a later stage of LDL oxidation, cholesterol or 7-ketocholesteryl esters of 9-oxononanoate derived from cholesteryl linoleate, are detected as the most abundant fraction of oxidized cholesteryl linoleate (Kamido *et al.*, 1992; Kamido *et al.*, 1995; Hoppe *et al.*, 1997). As a result of oxidation, a large number of oxidative structures are literally generated.

Chemically modified LDLs, such as MDA-modified LDL, acetylated LDL and $Cu^{2+}$-mediated oxLDL, were extensively examined as experimental models of denatured LDL to study atherogenic mechanisms. Among these models, trace amounts of $Cu^{2+}$ can induce LDL oxidation, resulting in highly reproducible LDL damage (Kleinveld *et al.*, 1992). This process leads to an oxLDL structure that shares many functional properties with the LDL oxidized by cells or to oxLDL extracted from arterial atherosclerotic plaques. Incubation of LDL with several different types of cells, or with $Cu^{2+}$ even in the absence of cells, results in an oxLDL structure with similar properties (Parthasarathy *et al.*, 1990). There is general consensus that $Cu^{2+}$-oxLDL is a relevant autoantigen because the oxLDL found in atheromatous lesions and the oxLDL extracted from atherosclerotic lesions, exhibited similar physicochemical and immunological properties to the $Cu^{2+}$-oxLDL (Yla-Herttuala *et al.*, 1989). Thus, $Cu^{2+}$-mediated oxLDL seems to be a more suitable model for physiological LDL rather than other chemically modified LDL, such as MDA-LDL. *In vivo*, LDL might be alternatively oxidized by released $Cu^{2+}$ from ceruloplasmin, the major copper-containing component of mammalian plasma (Ehrenwald *et al.*, 1994; Lamb and Leake, 1994).

### A POTENTIAL METABOLIC FORM OF oxLDL (oxLDL/β2GPI COMPLEXES)

β2GPI is a 50 kDa single-chain polypeptide composed of 326 amino acid residues, arranged in 5 homologous repeats known as complement control protein domains. β2GPI's fifth domain contains a patch of positively charged amino acids that likely represents the binding region for phospholipids (Bouma *et al.*, 1999; Hoshino *et al.*, 2000). β2GPI binds strongly to negatively charged molecules, such as phospholipids, heparin and certain lipoproteins as well as to activated platelets and apoptotic cell membranes. This binding may aid the clearance of apototic cells from circulation (Inanc *et al.*, 1997).

Antiphospholipid antibodies such as aCL and LA, have been found in patients with SLE in association with venous and arterial thromboembolic manifestations, with pregnancy morbidity (Harris *et al.*, 1983; Hughes *et al.*, 1986). Subgroups of SLE patients with these clinical and serologic features were classified as having APS. It is now widely agreed that some phospholipid-binding plasma proteins are the relevant antigens for antiphospholipid antibodies. In 1990, two groups of investigators (McNeil *et al.*, 1990; Matsuura *et al.*, 1990) independently reported that autoimmune aCL antibodies were directed to cardiolipin/plasma cofactor (β2-GPI) complexes. We further reported that aCL recognize a neo-epitope on β2-GPI when adsorbed onto hydrophobic surfaces with a negative charge such as an oxygenated polystyrene plate (Matsuura *et al.*, 1994).

We recently demonstrated (Hasunuma *et al.*, 1997; Kobayashi *et al.*, 2001; Liu *et al.*, 2002; Kobayashi *et al.*, 2003) the specific interaction between $Cu^{2+}$-oxLDL and β2GPI by ELISA (β2GPI by ELISA and optical sensor) and optical biosensor (Fig. 2). Further, two oxLDL-derived lipid ligands specific for β2GPI, namely, oxLig-1 and oxLig-2, were identified as 7-ketocholesteryl-9-carboxynonanoate [9-oxo-9-(7-ketocholest-5-en-3β-yloxy) nonanoic acid (IUPAC)] and 7-ketocholesteryl-12-carboxy (keto) dodecanoate, respectively (Fig. 3). Cholesteryl linoleate present in LDL is a major core lipid and represents the candidate for a precursor of these ligands.

The initial *in vitro* interaction of $Cu^{2+}$-oxLDL with β2GPI is due to electrostatic interactions between ω-carboxyl functions and lysine residues of β2GPI and is reversible by $Mg^{2+}$ treatment. The interaction later progresses to a much more stable bond such as Schiff base formation with ω-aldehydes (Fig. 4). Interestingly, the negative charges of oxLDL generated by incubating with $Cu^{2+}$ were neutralized by the interaction with β2GPI (Fig. 5). As described later, we demonstrated the presence of stable oxLDL/β2GPI complexes in sera of patients with various chronic diseases, such as APS, SLE, chronic renal diseases and diabetes mellitus, as well. These complexes are occasionally present in APS and SLE patients as immune complexes with IgG anti-β2GPI antibodies (Kobayashi *et al.*, 2003). The strength of the bond formed and the neutralization of the charges by the complexes may contribute to their long-term presence in the blood stream.

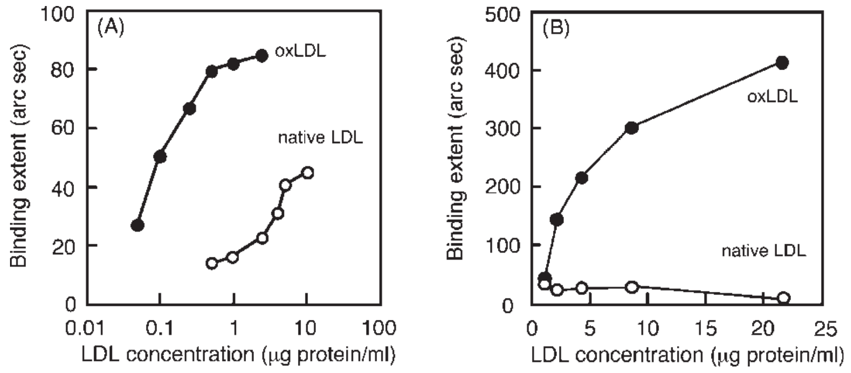

FIGURE 2 Molecular interactions among LDLs, β2GPI, and anti-β2GPI autoantibodies detected by optical biosensor (IAsys). (A) Native LDL or oxLDL binding to solid phase β2GPI. (B) Native LDL or oxLDL binding to solid phase WB-CAL-1 antibody (anti-β2GPI autoantibody) in the presence of β2GPI.

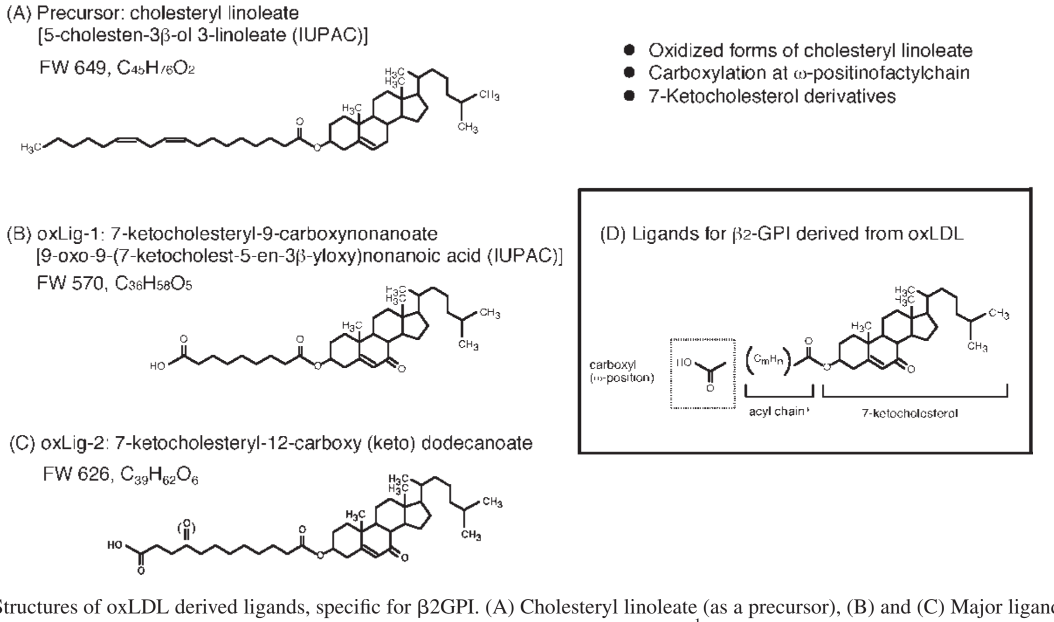

FIGURE 3 Structures of oxLDL derived ligands, specific for β2GPI. (A) Cholesteryl linoleate (as a precursor), (B) and (C) Major ligands for β2GPI (oxLig-1 and oxLig-2, respectively), (C) A common structure of the β2GPI ligands. oxLig-1: $^1$H-NMR (300.1 MHz, $CDCl_3$):$\delta = 5.71$ (s, 1 H, H-6), 4.78-4.69 (m, 1 H, H-3); $^{13}$C-NMR (75.5 MHz, $CDCl_3$): $\delta = 202.5$, 179.7, 173.4, 164.5, 127.1, 72.4, 55.2, 50.4, 50.2, 45.8, 43.5, 39.9, 38.7, 36.6, 36.1, 29.2, 28.9, 28.4, 25.3, 25.0, 24.2, 23.2, 23.0, 19.3, 17.7, 12.4; m/z (FD-MS): 571 $[(M + H)^+$, $C_{36}H_{59}O_5$ requires 571].

### IGG AUTOANTIBODY-MEDIATED PHAGOCYTOSIS OF oxLDL/β2GPI COMPLEXES

We first demonstrated in 1997 that the *in vitro* macrophage uptake of $^{125}$I-$Cu^{2+}$-oxLDL was significantly enhanced in the presence of β2GPI and IgG anti-β2GPI autoantibodies (Hasunuma *et al.*, 1997). Further, the macrophage uptake of liposomes containing β2GPI ligands (oxLig-1 and oxLig-2) was also enhanced confirming the previous results (Kobayashi *et al.*, 2001; Liu *et al.*, 2002; Kobayashi *et al.*, 2003). These findings indicate that IgG anti-β2GPI autoantibodies may be atherogenic. The *in vivo* oxLDL uptake is likely mediated by Fcγ receptors rather than by scavenger receptors (Fig. 1B). In contrast, most recent reports indicated that natural antibodies (mainly of the IgM class) derived from hyperlipidemic mice reduced the incidence of atherosclerosis in experimental models (Chang *et al.*, 1999; Horkko *et al.*, 1999; Binder *et al.*, 2003; Rose and Afanasyeva, 2003). Because, Fcμ receptors have poor phagocytic properties, possibly making IgM class of autoantibodies and/or natural antibodies anti-atherogenic (or protective).

### AUTOIMMUNITY AND ATHEROSCLEROSIS

Antiphospholipid antibodies, i.e. β2GPI-dependent aCL, anti-β2GPI and anti-prothrombin antibodies have been associated with several forms of cardiovascular diseases

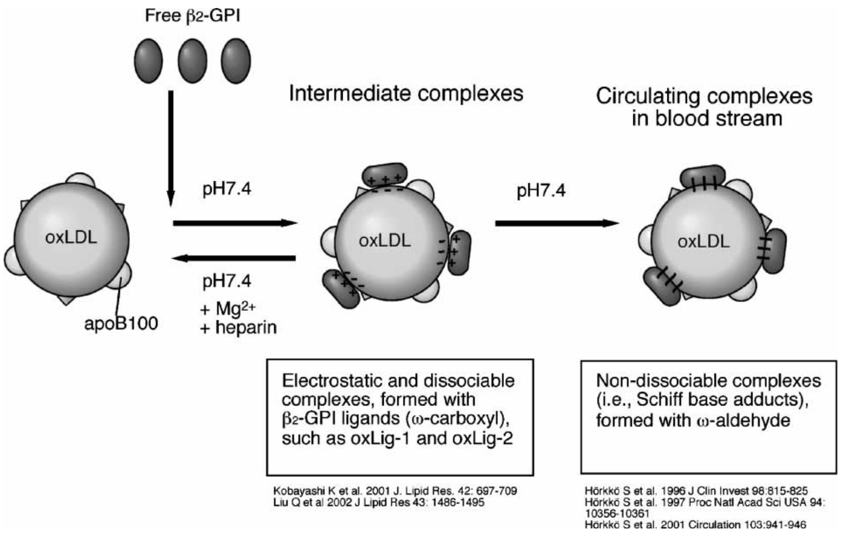

FIGURE 4 Mechanism of complex formation between oxLDL and β2GPI.

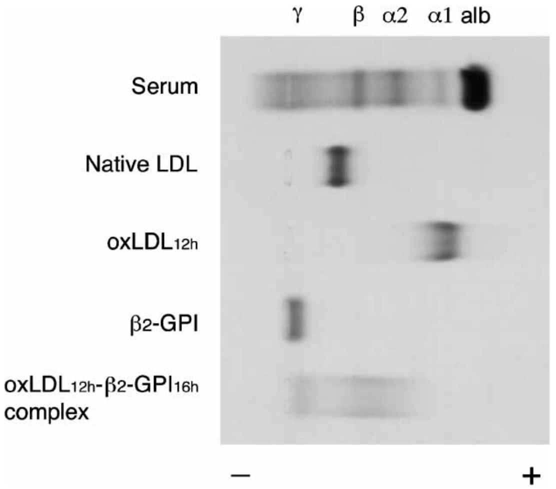

FIGURE 5 Agarose electrophoresis of oxLDL/β2GPI complexes. oxLDL12 h: LDL was oxidized by incubating with Cu2+ion for 12 h. Oxidized $LDL_{12\,h}$-β2-$GPI_{16\,h}$: complexes prepared by incubating oxLDL12 and β2GPI for 16 h at 37 °C.

such as myocardial infarction, stroke, carotid stenosis, etc. (Vaarala, 1998; Brey *et al.*, 2001). The finding that some aCL from patients with APS were capable of cross-reacting with oxLDL (Vaarala *et al.*, 1993), suggested that antiphospholipid antibodies might promote atherosclerosis. The plasma protein β2GPI, now regarded as the relevant antigenic target for antiphospholipid antibodies, has been found in atherosclerotic lesions together with immunoreactive CD4 lymphocytes (George *et al.*, 1999). These observations not only support the hypothesis of an autoimmune mechanism in atherosclerosis but also may indicate a role for antiphospholipid antibodies in this process. It is possible that systemic autoimmune diseases may cause vascular inflammation resulting in oxidation of LDL and these changes along with a series of biochemical events and complex molecular interactions may produce an autoantibody response leading to atherosclerosis.

The premature (or accelerated) development of atherosclerosis has been recently recognized in patients with systemic autoimmune diseases (Ward, 1999; Aranow and Ginzler, 2000; Van Doornum *et al.*, 2002). The traditional risk factors for atherosclerosis as well as autoimmune disease treatments (i.e. steroids) failed to account for these changes (Esdaile *et al.*, 2001). Today's SLE survival rate (>80% over a 10-year follow-up) may have uncovered hidden causes of mortality and morbidity. Systemic lupus erythematosus mortality rates due to cardiovascular disease have surpassed that from SLE disease itself or from complications such as infections in most studies (Schattner and Liang, 2003). In addition, abnormal myocardial perfusion results by Tc99m emission tomography have been reported in about 43% of asymptomatic SLE patients and increased carotid intima medial thickness (IMT) with atherosclerotic plaques have also been demonstrated by B-mode ultrasound in over 33% of these patients. Autoantibodies against oxLDL, phospholipids and Lp(a), have been proposed as alternative mechanisms as well as certain biochemical and genetic abnormalities (Lockshin *et al.*, 2001). Although venous thromboembolic complications are the most common clinical finding in APS patients (Harris *et al.*, 1986; Ginsburg *et al.*, 1992; Bick and Baker, 1999), about 25% of the APS patients enrolled into a European cohort of 1,000 patients presented an arterial thrombotic event (myocardial infarction, cerebrovascular accident, angina, etc.) as the initial clinical manifestation. When all the arterial thrombotic events were considered, up to 31% of the patients presented these complications (Cervera *et al.*, 2002).

### ANTIPHOSPHOLIPID SYNDROME AND ATHEROSCLEROSIS

Elevated serum levels of antiphospholipid antibodies along with thrombotic events of both the venous and arterial vasculature, or with pregnancy morbidity represent the major features of APS. APS may be present in the context of a systemic autoimmune disorder i.e. SLE (Hughes *et al.*, 1986). Antiphospholipid antibodies appear to increase the risk of thrombosis by at least two-fold in the context of an autoimmune disease (Wahl *et al.*, 1997). In APS, recurrence rates of up to 30% for thrombosis with a mortality of up to 10% over a 10-year follow-up period have been reported (Shah *et al.*, 1998). Antiphospholipid syndrome is the most common cause of acquired hypercoagulability in the general population.

Antiphospholipid antibodies are a heterogeneous group of autoantibodies. These antibodies are characterized by their reactivity to negatively charged phospholipids, phospholipid/protein complexes and certain proteins presented on suitable surfaces (i.e. activated cell membranes, oxygenated polystyrene) (Matsuura *et al.*, 1994; Roubey, 1994). Several plasma proteins that participate in coagulation and interact with phospholipids have been described as antiphospholipid cofactors, i.e. β2GPI, prothrombin and annexin V. β2GPI has been shown to be a relevant antigenic target for antiphospholipid antibodies (McNeil *et al.*, 1990; Matsuura *et al.*, 1990; Matsuura *et al.*, 1992; Matsuura *et al.*, 1994). Binding of antiphospholipid antibodies to β2GPI may interfere with the anticoagulant functions promoting thrombosis. Anti-β2GPI antibodies have been reported as more specific for thrombosis and APS than aCL antibodies (Tsutsumi *et al.*, 1996) and recent prospective studies have shown that aCL antibodies, particularly β2GPI-dependent, or anti-β2GPI antibodies are important predictors for arterial thrombosis (myocardial infarction and stroke) in men (Vaarala, 1998; Brey *et al.*, 2001; Lopez *et al.*, 2004). These studies suggest that β2GPI and anti-β2GPI antibodies play a pathogenic role in the development of thrombosis, particularly in arterial thrombosis (atherosclerosis) in SLE and APS patients.

We have demonstrated that oxLDL, not native LDL, binds β2GPI, initially forming dissociable electrostatic complexes followed by more stable complexes bound by covalent interactions (Kobayashi *et al.*, 2003). In addition, we also reported that oxidation of LDL is a common occurrence in both APS and SLE patients without APS and have demonstrated stable oxLDL/β2GPI complexes in more than 60% of these patients. However, serum levels of these complexes were not associated with the activity of SLE or with any major clinical manifestation of APS (Lopez *et al.*, 2004). Although it can be hypothesized that this interaction might be related to chronic inflammation of the vasculature that occurs in autoimmune patients, the mechanism(s) for the increased oxidation of LDL are not fully understood. Significantly high serum levels of oxLDL/β2GPI complexes fluctuated widely at different time intervals over a 12-month follow-up from 6 SLE patients. This suggests that oxidation and formation of complexes are very active processes under unknown regulatory mechanism(s). Stable oxLDL/β2GPI complexes may be clinically relevant as they have been implicated as atherogenic autoantigens and their presence may represent a risk factor or an indirect but significant contributor for thrombosis and atherosclerosis in patients with an autoimmune background (Kobayashi *et al.*, 2003).

We have also measured serum levels of IgG anti-oxLig-1 (oxLDL)/β2GPI autoantibodies in patients with APS and evaluated their association with the major clinical manifestations of APS (Kobayashi *et al.*, 2003; Lopez *et al.*, 2003). IgG autoantibodies against oxLig-1/β2GPI were significantly elevated in about 40% of patients with APS as compared to SLE patients without APS. Further, these antibodies showed a stronger correlation with arterial thrombosis than venous thrombosis and pregnancy morbidity. Anti-oxLig-1/β2GPI antibodies were present in 3 of 4 SLE patients with active disease followed over a 12-month period, while 2 patients with inactive disease and oxLDL/β2GPI complexes did not have these antibodies (Lopez *et al.*, 2004). The coexistence of oxLig-1/β2GPI autoantibodies with oxLDL/β2GPI complexes, suggest that these two elements interact perhaps forming circulating immune complexes (oxLDL/β2GPI/antibody). Actually, we detected such immune complexes in serum samples of SLE and/or APS in the most recent study (Kobayashi *et al.*, 2003). This observation along with the increased macrophage uptake of oxLDL/β2GPI complexes in the presence of anti-oxLig-1/β2GPI antibodies (Hasunuma *et al.*, 1997; Tinahones *et al.*, 1998; Kobayashi *et al.*, 2001; Liu *et al.*, 2002), provide a possible explanation for the accelerated development of atherosclerosis in autoimmune patients.

### CONCLUSION

Current atherosclerosis research at different levels i.e. histopathological, biochemical and immunological continues to unravel the complex nature of this process. New factors are being investigated and unique interactions among diverse factors described, all resulting in a better understanding of atherogenic mechanism(s). In addition to the oxidative modification of LDL, the action of local soluble mediators of inflammation and chemotactic factors and the accumulation of immune cells (macrophages and T lymphocytes) in response to various external and/or internal stimuli, an active humoral immune response has been recently incorporated into the process. A significant role of antiphospholipid antibodies in the development of atherosclerosis was proposed when some groups reported in SLE patients the presence of antibodies to oxLDL, some with cross-reactivity with aCL antibodies, as well as the localization of β2GPI in the human atherosclerotic plaques. The interaction between oxLDL and β2GPI to form circulating complexes strongly suggests that this complex is an atheroantigen. This interaction has been further characterized and the oxLDL-derived ligand (oxLig-1) specific for β2GPI has been identified and synthesized. Antiphospholipid syndrome patients may produce antibodies to this complex and the resulting circulating immune complexes trigger atherosclerotic changes. The physiologic relevance of this finding has been demonstrated *in vitro* by the enhanced macrophage uptake of oxLDL/β2GPI/antibody complexes. The antibodies used in these experiments were aCL antibodies β2GPI-dependent or anti-β2GPI antibodies. The possible participation of Fcγ receptors in the increased macrophage uptake of oxLDL-containing complexes seems to be particularly important in the development of foam cells and atherosclerotic plaques in the intima of arteries.

Stable and possibly pathogenic oxLDL/β2GPI complexes were demonstrated in the serum of both SLE and APS patients, but anti-oxLDL/β2GPI (oxLig-1) antibodies seem to be restricted to SLE patients with APS or primary APS. Further, the association of these antibodies with arterial thrombosis was stronger than with venous thrombosis in these patients. At this point, these results should be interpreted in the context of an autoimmune disease. We have also detected oxLDL/β2GPI complexes in patients with syphilis but not in rheumatoid arthritis, indicating that oxidation of LDL and the formation of complexes with β2GPI is not restricted to SLE. In contrast, both patient groups, syphilis and rheumatoid arthritis, did not develop anti-oxLDL/β2GPI (oxLig-1) antibodies. These antibodies seem to be restricted to SLE and APS and we hypothesize that the presence of these antibodies accelerates the development of atherosclerosis (Grainger and Bethell, 2002).

### *References*

Aranow, C. and Ginzler, E.M. (2000) "Epidemiology of cardiovascular disease in systemic lupus erythematosus", *Lupus* **9**, 166–169.

Bick, R.L. and Baker, W.F. (1999) "Antiphospholipid syndrome and thrombosis", *Semin. Thromb. Hemost.* **25**, 333–350.

Binder, C.J., Horkko, S., Dewan, A., Chang, M.K., Kieu, E.P., Goodyear, C.S., Shaw, P.X., Palinski, W., Witztum, J.L. and Silverman, G.J. (2003) "Pneumococcal vaccination decreases atherosclerotic lesion formation: molecular mimicry between *Streptococcus pneumoniae* and oxidized LDL", *Nat. Med.* **9**, 736–743.

Bouma, B., de Groot, P.G., van den Elsen, J.M., Ravelli, R.B., Schouten, A., Simmelink, M.J., Derksen, R.H., Kroon, J. and Gros, P. (1999)

"Adhesion mechanism of human β2-glycoprotein I to phospholipids based on its crystal structure", *EMBO J.* **18**, 5166–5174.

Brey, R.L., Abbott, R.D., Curb, J.D., Sharp, D.S., Ross, G.W., Stallworth, C.L. and Kittner, S.J. (2001) "β2-glycoprotein I dependent anticardiolipin antibodies and the risk of ischemic stroke and myocardial infarction", *Stroke* **32**, 1701–1706.

Brown, M.S. and Goldstein, J.L. (1985) "Scavenger cell receptor shared", *Nature* **316**, 680–681.

Brown, A.J. and Jessup, W. (1999) "Oxysterols and atherosclerosis", *Atherosclerosis* **142**, 1–28.

Brown, A.J., Leong, S.L., Dean, R.T. and Jessup, W. (1997) "7-Hydroperoxycholesterol and its products in oxidized low density lipoprotein and human atherosclerotic plaque", *J. Lipid. Res.* **38**, 1730–1745.

Cervera, R., Piette, J.C., Font, J., Khamashta, M.A., Shoenfeld, Y., Camps, M.T., Jacobsen, S., Lakos, G., Tincani, A., Kontopoulou-Griva, I., Galeazzi, M., Meroni, P.L., Derksen, R.H., de Groot, P.G., Gromnica-Ihle, E., Baleva, M., Mosca, M., Bombardieri, S., Houssiau, F., Gris, J.C., Quere, I., Hachulla, E., Vasconcelos, C., Roch, B., Fernandez-Nebro, A., Boffa, M.C., Hughes, G.R., Ingelmo, M. and Euro-Phospholipid Project Group. (2002) "Antiphospholipid syndrome. Clinical and immunologic manifestations and patterns of disease expression in a cohort of 1000 patients", *Arthritis Rheum.* **46**, 1019–1027.

Chang, M.K., Bergmark, C., Laurila, A., Horkko, S., Han, K.H., Friedman, P., Dennis, E.A. and Witztum, J.L. (1999) "Monoclonal antibodies against oxidized low-density lipoprotein bind to apoptotic cells and inhibit their phagocytosis by elicited macrophages: evidence that oxidation-specific epitopes mediate macrophage recognition", *Proc. Natl Acad. Sci. USA* **96**, 6353–6358.

Cushing, S.D., Berliner, J.A., Valente, A.J., Territo, M.C., Navab, M., Parhami, F., Gerrity, R., Schwartz, C.J. and Fogelman, A.M. (1990) "Minimally modified low density lipoprotein induces monocyte chemotactic protein 1 in human endothelial cells and smooth muscle cells", *Proc. Natl Acad. Sci. USA* **87**, 5134–5138.

Ehrenwald, E., Chisolm, G.M. and Fox, P.L. (1994) "Intact ceruloplasmin oxidatively modifies low density lipoprotein", *J. Clin. Invest.* **93**, 1493–1501.

Elomaa, O., Kangas, M., Sahlberg, C., Tuukkanen, J., Sormunen, R., Liakka, A., Thesleff, I., Kraal, G. and Tryggvason, K. (1995) "Cloning of a novel bacteria-binding receptor structurally related to scavenger receptors and expressed in a subset of macrophages", *Cell* **80**, 603–609.

Elomaa, O., Sankala, M., Pikkarainen, T., Bergmann, U., Tuuttila, A., Raatikainen-Ahokas, A., Sariola, H. and Tryggvason, K. (1998) "Structure of the human macrophage MARCO receptor and characterization of its bacteria-binding region", *J. Biol. Chem.* **273**, 4530–4538.

Esdaile, J.M., Abrahamowicz, M., Grodzicky, T., Li, Y., Panaritis, C., du Berger, R., Cote, R., Grover, S.A., Fortin, P.R., Clarke, A.E. and Senecal, J.L. (2001) "Traditional Framingham risk factors fail to fully account for accelerated atherosclerosis in systemic lupus erythematosus", *Arthritis Rheum.* **44**, 2331–2337.

Esterbauer, H., Jurgens, G., Quehenberger, O. and Koller, E. (1987) "Autoxidation of human low density lipoprotein: loss of polyunsaturated fatty acids and vitamin E and generation of aldehydes", *J. Lipid. Res.* **28**, 495–509.

Fong, L.G., Parthasarathy, S., Witztum, J.L. and Steinberg, D. (1987) "Nonenzymatic oxidative cleavage of peptide bonds in apoprotein B-100", *J. Lipid. Res.* **28**, 1466–1477.

Frostegard, J., Wu, R., Haegerstrand, A., Patarroyo, M., Lefvert, A.K. and Nilsson, J. (1993) "Mononuclear leukocytes exposed to oxidized low density lipoprotein secrete a factor that stimulates endothelial cells to express adhesion molecules", *Atherosclerosis* **103**, 213–219.

Galli, M., Comfurius, P., Maassen, C., Hemker, H.C., de Baets, M.H., Van Breda-Vriesman, P.J.C., Barbui, T., Zwaal, R.F.A. and Bevers, E.M. (1990) "Anticardiolipin antibodies (ACA) directed not to cardiolipin but to a plasma protein cofactor", *Lancet* **335**, 1544–1547.

George, J., Harats, D., Gilburd, B., Afek, A., Levy, Y., Schneiderman, J., Barshack, I., Kopolovic, J. and Shoenfeld, Y. (1999) "Immunolocalization of β2-glycoprotein I (apolipoprotein H) to human atherosclerotic plaques: potential implications for lesion progression", *Circulation* **99**, 2227–2230.

Ginsburg, K.S., Liang, M.H., Newcomer, L., Goldhaber, S.Z., Schur, P.H., Hennekens, C.H. and Stampfer, M.J. (1992) "Anticardiolipin antibodies and the risk for ischemic stroke and venous thrombosis", *Ann. Intern. Med.* **117**, 997–1002.

Goldstein, J.L., Ho, Y.K., Basu, S.K. and Brown, M.S. (1979) "Binding site on macrophages that mediates uptake and degradation of acetylated low density lipoprotein, producing massive cholesterol deposition", *Proc. Natl Acad. Sci. USA* **76**, 333–337.

Harris, E.N., Gharavi, A.E., Boey, M.L., Patel, B.M., Mackworth-Young, C.G., Loizou, S. and Hughes, G.R.V. (1983) "Anticardiolipin antibodies: detection by radioimmunoassay and association with thrombosis in systemic lupus erythematosus", *Lancet* **2**, 1211–1214.

Harris, E.N., Chan, J.K.H., Asherson, R.A. and Hughes, G.R.V. (1986) "Thrombosis, recurrent fetal loss and thrombocytopenia-predictive value of the anticardiolipin antibody test", *Arch. Intern. Med.* **146**, 2153–2156.

Hasunuma, Y., Matsuura, E., Makita, Z., Katahira, T., Nishi, S. and Koike, T. (1997) "Involvement of β2-glycoprotein I and anti-cardiolipin antibodies in oxidatively modified low-density lipoprotein uptake by macrophages", *Clin. Exp. Immunol.* **107**, 569–573.

van Heek, M., Schmitt, D., Toren, P., Cathcart, M.K. and DiCorleto, P.E. (1998) "Cholesteryl hydroperoxyoctadecadienoate from oxidized low density lipoprotein inactivated platelet-derived growth factor", *J. Biol. Chem.* **273**, 19405–19410.

Heinecke, J.W. (1997) "Mechanisms of oxidative damage of low density lipoprotein in human atherosclerosis", *Curr. Opin. Lipid.* **8**, 268–274.

Hoppe, G., Ravandi, A., Herrera, D., Kuksis, A. and Hoff, H.F. (1997) "Oxidation products of cholesteryl linoleate are resistant to hydrolysis in macrophages, form complexes with proteins, and are present in human atherosclerotic lesions", *J. Lipid Res.* **38**, 1347–1360.

Horkko, S., Bird, D.A., Miller, E., Itabe, H., Leitinger, N., Subbanagounder, G., Berliner, J.A., Friedman, P., Dennis, E.A., Curtiss, L.K., Palinski, W. and Witztum, J.L. (1999) "Monoclonal autoantibodies specific for oxidized phospholipids or oxidized phospholipid-protein adducts inhibit macrophage uptake of oxidized low-density lipoproteins", *J. Clin. Invest.* **103**, 117–128.

Hoshino, M., Hagihara, Y., Nishii, I., Yamazaki, T., Kato, H. and Goto, Y. (2000) "Identification of the phospholipid-binding site of human β2-glycoprotein I domain V by heteronuclear magnetic resonance", *J. Mol. Biol.* **304**, 927–939.

Hughes, G.R.V., Harris, E.N. and Gharavi, A.E. (1986) "The anti-cardiolipin syndrome", *J. Rheumatol.* **13**, 486–489.

Inanc, M., Radway-Bright, E.L. and Isenberg, D.A. (1997) "β2-glycoprotein I and anti-β2-glycoprotein I antibodies: where are we now?", *Br. J. Rheumatol.* **36**, 1247–1257.

Kamido, H., Kuksis, A., Marai, L. and Myher, J.J. (1992) "Identification of cholesterol-bound aldehydes in copper-oxidized low density lipoprotein", *FEBS Lett.* **304**, 269–272.

Kamido, H., Kuksis, A., Marai, L. and Myher, J.J. (1995) "Lipid ester-bound aldehydes among copper-*catalyzed* peroxidation products of human plasma lipoproteins", *J. Lipid. Res.* **36**, 1876–1886.

Kleinveld, H.A., Hak-Lemmers, H.L.M., Stalenhoef, A.F.H. and Demacker, P.N.M. (1992) "Improved measurement of low-density-lipoprotein susceptibility to copper-induced oxidation: application of a short procedure for isolating low-density lipoprotein", *Clin. Chem.* **38**, 2066–2072.

Kobayashi, K., Matsuura, E., Liu, Q., Furukawa, J., Kaihara, K., Inagaki, J., Atsumi, T., Sakairi, N., Yasuda, T., Voelker, D.R. and Koike, T. (2001) "A specific ligand for β2-glycoprotein I mediates autoantibody-dependent uptake of oxidized low density lipoprotein by macrophages", *J. Lipid. Res.* **42**, 697–709.

Kobayashi, K., Kishi, M., Atsumi, T., Bertolaccini, M.L., Makino, H., Sakairi, N., Yamamoto, I., Yasuda, T., Khamashta, M.A., Hughes, G.R.V., Koike, T., Voelker, D.R. and Matsuura, E. (2003) "Circulating oxidized LDL forms complexes with β2-glycoprotein I: implication as an atherogenic autoantigen", *J. Lipid. Res.* **44**, 716–726.

Kodama, T., Reddy, P., Kishimoto, C. and Krieger, M. (1988) "Purification and characterization of a bovine acetyl low density lipoprotein receptor", *Proc. Natl Acad. Sci. USA* **85**, 9238–9242.

Kodama, T., Freeman, M., Rohrer, L., Zabrecky, J., Matsudaira, P. and Krieger, M. (1990) "Type I macrophage scavenger receptor contains alpha-helical and collagen-like coiled coils", *Nature* **343**, 531–535.

Kritharides, L., Jessup, W., Gifford, J. and Dean, R.T. (1993) "A method for defining the stages of low density lipoprotein oxidation by the separation of cholesterol- and cholesteryl ester-oxidation products using HPLC", *Anal. Biochem.* **213**, 79–89.

Lamb, D. and Leake, D.S. (1994) "Acidic pH enables caeruloplasmin to catalyse the modification of low-density lipoprotein", *FEBS Lett.* **338**, 122–126.

Liu, Q., Kobayashi, K., Inagaki, J., Sakairi, N., Iwado, A., Yasuda, T., Koike, T., Voelker, D.R. and Matsuura, E. (2002) "ω-carboxyl variants of 7-ketocholesteryl esters are ligands for β2-glycoprotein I and mediate antibody-dependent uptake of oxidized LDL by macrophages", *J. Lipid. Res.* **43**, 1486–1495.

Lockshin, M.D., Salmon, J.E. and Roman, M.J. (2001) "Atherosclerosis and Lupus: A work in progress", *Arthritis Rheum.* **44**, 2215–2217.

Lopez, D., Kobayashi, K., Merrill, J.T., Matsuura, E. and Lopez, L.R. (2003) "IgG Autoantibodies against β2-glycoprotein I complexed with a lipid ligand derived from oxidized low-density lipoprotein are associated with arterial thrombosis in antiphospholipid syndrome", *Clin. Dev. Immunol.* **10**, 203–211.

Lopez, L.R., Dier, K.J., Lopez, D., Merrill, J.T. and Fink, C.A. (2004) "Anti-β2-glycoprotein I and antiphosphatidylserine antibodies are predictors of arterial thrombosis in patients with antiphospholipid syndrome", *Am. J. Clin. Pathol.* **121**, 142–149.

Lopez, D., Garcia-Valladares, I., Palafox-Sanchez, C., Garcia De La Torre, I., Kobayashi, K., Matsuura, E. and Lopez, L.R. (2004) "Oxidized low-density lipoprotein/β2-glycoprotein I complexes and autoantibodies to oxLig-1/β2-glycoprotein I in patients with systemic lupus erythematosus and antiphospholipid syndrome", *Am. J. Clin. Pathol.* **121**, 426–436.

Mach, F., Schonbeck, U., Sukhova, G.K., Atkinson, E. and Libby, P. (1998) "Reduction of atherosclerosis in mice by inhibition of CD40 signalling", *Nature* **394**, 200–203.

Matsuura, E., Igarashi, Y., Fujimoto, M., Ichikawa, K. and Koike, T. (1990) "Anticardiolipin cofactor(s) and differential diagnosis of autoimmune disease", *Lancet* **336**, 177–178.

Matsuura, E., Igarashi, Y., Fujimoto, M., Ichikawa, K., Suzuki, T., Sumida, T., Yasuda, T. and Koike, T. (1992) "Heterogeneity of anticardiolipin antibodies defined by the anticardiolipin cofactor", *J. Immunol.* **148**, 3885–3891.

Matsuura, E., Igarashi, Y., Yasuda, T., Triplett, D.A. and Koike, T. (1994) "Anticardiolipin antibodies recognize β2-glycoprotein I structure altered by interacting with an oxygen modified solid phase surface", *J. Exp. Med.* **179**, 457–462.

McNeil, H.P., Simpson, R.J., Chesterman, C.N. and Krilis, S.A. (1990) "Anti-phospholipid antibodies are directed against a complex antigen that includes a lipid-binding inhibitor of coagulation: β2-glycoprotein I (apolipoprotein H)", *Proc. Natl Acad. Sci. USA* **87**, 4120–4124.

Minami, M., Kume, N., Shimaoka, T., Kataoka, H., Hayashida, K., Akiyama, Y., Nagata, I., Ando, K., Nobuyoshi, M., Hanyuu, M., Komeda, M., Yonehara, S. and Kita, T. (2001) "Expression of SR-PSOX, a novel cell-surface scavenger receptor for phosphatidylserine and oxidized LDL in human atherosclerotic lesions", *Arterioscler. Thromb. Vasc. Biol.* **21**, 1796–1800.

Nagy, L., Tontonoz, P., Alvarez, J.G., Chen, H. and Evans, R.M. (1998) "Oxidized LDL regulates macrophage gene expression through ligand activation of PPAR (γ)", *Cell* **93**, 229–240.

Nakata, A., Miyagawa, J., Yamashita, S., Nishida, M., Tamura, R., Yamamori, K., Nakamura, T., Nozaki, S., Kameda-Takemura, K., Kawata, S., Taniguchi, N., Higashiyama, S. and Matsuzawa, Y. (1996) "Localization of heparin-binding epidermal growth factor-like growth factor in human coronary arteries. Possible roles of HB-EGF in the formation of coronary atherosclerosis", *Circulation* **94**, 2778–2786.

Parthasarathy, S., Fong, L.G., Quinn, M.T. and Steinberg, D. (1990) "Oxidative modification of LDL: comparison between cell-mediated and copper-mediated modification", *Eur. Heart. J.* **11**, 83–87.

Rajavashisth, T.B., Andalibi, A., Territo, M.C., Berliner, J.A., Navab, M., Fogelman, A.M. and Lusis, A.J. (1990) "Induction of endothelial cell expression of granulocyte and macrophage colony-stimulating factors by modified low-density lipoproteins", *Nature* **344**, 254–257.

Ramprasad, M.P., Fischer, W., Witztum, J.L., Sambrano, G.R., Quehenberger, O. and Steinberg, D. (1995) "The 94- to 97 kDa mouse macrophage membrane protein that recognizes oxidized low density lipoprotein and phosphatidylserine-rich liposomes is identical to macrosialin, the mouse homologue of human CD68", *Proc. Natl Acad. Sci. USA* **92**, 9580–9584.

Rigotti, A., Acton, S.L. and Krieger, M. (1995) "The class B scavenger receptors SR-BI and CD36 are receptors for anionic phospholipids", *J. Biol. Chem.* **270**, 16221–16224.

Romero, F.I., Amengual, O., Atsumi, T., Khamashta, M.A., Tinahones, F.J. and Hughes, G.R.V. (1998) "Arterial disease in lupus and secondary antiphospholipid syndrome: association with anti-β2-glycoprotein I antibodies but not with antibodies against oxidized low-density lipoprotein", *Br. J. Rheumatol.* **37**, 883–888.

Rose, N. and Afanasyeva, M. (2003) "Autoimmunity: busting the atheorsclerotic plaque", *Nat. Med.* **9**, 641–642.

Roubey, R.A. (1994) "Autoantibodies to phospholipid-binding plasma proteins: a new view of lupus anticoagulants and other "anti-phospholipid" autoantibodies", *Blood* **84**, 2854–2867.

Sambrano, G.R. and Steinberg, D. (1995) "Recognition of oxidatively damaged and apoptotic cells by an oxidized low density lipoprotein receptor on mouse peritoneal macrophages: role of membrane phosphatidylserine", *Proc. Natl Acad. Sci. USA* **92**, 1396–1400.

Sawamura, T., Kume, N., Aoyama, T., Moriwaki, H., Hoshikawa, H., Aiba, Y., Tanaka, T., Miwa, S., Katsura, Y., Kita, T. and Masaki, T. (1997) "An endothelial receptor for oxidized low-density lipoprotein", *Nature* **386**, 73–77.

Schattner, A. and Liang, M.H. (2003) "The cardiovascular burden of lupus; a complex challenge", *Arch. Intern. Med.* **163**, 1507–1510.

Shah, N.M., Khamashta, M.A., Atsumi, T. and Hughes, G.R. (1998) "Outcome of patients with anticardiolipin antibodies: a 10 year follow-up of 52 patients", *Lupus* **7**, 3–6.

Shimaoka, T., Kume, N., Minami, M., Hayashida, K., Kataoka, H., Kita, T. and Yonehara, S. (2000) "Molecular cloning of a novel scavenger receptor for oxidized low density lipoprotein, SR-PSOX, on macrophages", *J. Biol. Chem.* **275**, 40663–40666.

Steinberg, D., Parthasarathy, S., Carew, T.E., Khoo, J.C. and Witztum, J.L. (1989) "Beyond cholesterol. Modifications of low-density lipoprotein that increase its atherogenicity", *N. Engl. J. Med.* **320**, 915–924.

Steinberg, D. (1997) "Low density lipoprotein oxidation and its pathobiological significance", *J. Biol. Chem.* **272**, 20963–20966.

Steinbrecher, U.P., Parthasarathy, S., Leake, D.S., Witztum, J.L. and Steinberg, D. (1984) "Modification of low density lipoprotein by endothelial cells involves lipid peroxidation and degradation of low density lipoprotein phospholipids", *Proc. Natl Acad. Sci. USA* **81**, 3883–3887.

Tinahones, F.J., Cuadrado, M.J., Khamashta, M.A., Mujic, F., Gomez-Zumaquero, J.M., Collantes, E. and Hughes, G.R.V. (1998) "Lack of cross reaction between antibodies to β2-glycoprotein-I and oxidized low-density lipoprotein in patients with antiphospholipid syndrome", *Br. J. Rheumatol.*, 746–749.

Tsutsumi, A., Matsuura, E., Ichikawa, K., Fujisaku, A., Mukai, M., Kobayashi, S. and Koike, T. (1996) "Antibodies to β2-glycoprotein I and clinical manifestations in patients with systemic lupus erythematosus", *Arthritis Rheum.* **39**, 1466–1474.

Vaarala, O., Alfthan, G., Jauhiainen, M., Leirisalo-Repo, M., Aho, K. and Palosuo, T. (1993) "Crossreaction between antibodies to oxidised low-density lipoprotein and to cardiolipin in systemic lupus erythematosus", *Lancet* **341**, 923–925.

Vaarala, O. (1998) "Antiphospholipid antibodies in myocardial infarction", *Lupus* **7**, S132–S134.

Van Doornum, S., McColl, G. and Wicks, I.P. (2002) "Accelerated atherosclerosis. An extraarticular feature of rheumatoid arthritis?", *Arthritis Rheum.* **46**, 862–873.

Wahl, D.G., Guillemin, F., de Maistre, E., Perret, C., Lecompte, T. and Thibaut, G. (1997) "Risk for venous thrombosis related to antiphospholipid antibodies in systemic lupus erythematosus: a meta analysis", *Lupus* **6**, 467–473.

Ward, M.M. (1999) "Premature morbidity from cardiovascular and cerebrovascular diseases in women with systemic lupus erythematosus", *Arthritis Rheum.* **42**, 338–346.

Weidtmann, A., Scheithe, R., Hrboticky, N., Pietsch, A., Lorenz, R. and Siess, W. (1995) "Mildly oxidized LDL induces platelet aggregation through activation of phsopholipase A2", *Arterioscler. Thromb. Vasc. Biol.* **15**, 1131–1138.

Yamamoto, T., Davis, C.G., Brown, M.S., Schneider, W.J., Casey, M.L., Goldstein, J.L. and Russell, D.W. (1984) "The human LDL receptor: a cysteine-rich protein with multiple Alu sequences in its mRNA", *Cell* **39**, 27–38.

Yla-Herttuala, S., Palinski, W., Rosenfeld, M.E., Parthasarathy, S., Carew, T.E., Butler, S., Witztum, J.L. and Steinberg, D. (1989) "Evidence for presence of oxidatively modified low density lipoprotein in atherosclerotic lesions of rabbit and man", *J. Clin. Invest.* **85**, 1086–1095.

